# Genome reassembly with high-throughput sequencing data

**DOI:** 10.1186/1471-2164-14-S1-S8

**Published:** 2013-01-21

**Authors:** Nathaniel Parrish, Benjamin Sudakov, Eleazar Eskin

**Affiliations:** 1 Department of Computer Science, University of California Los Angeles, Los Angeles, California, USA; 2 Department of Mathematics, University of California Los Angeles, Los Angeles, California, USA

## Abstract

**Motivation:**

Recent studies in genomics have highlighted the significance of structural variation in determining individual variation. Current methods for identifying structural variation, however, are predominantly focused on either assembling whole genomes from scratch, or identifying the relatively small changes between a genome and a reference sequence. While significant progress has been made in recent years on both *de novo *assembly and resequencing (read mapping) methods, few attempts have been made to bridge the gap between them.

**Results:**

In this paper, we present a computational method for incorporating a reference sequence into an assembly algorithm. We propose a novel graph construction that builds upon the well-known de Bruijn graph to incorporate the reference, and describe a simple algorithm, based on iterative message passing, which uses this information to significantly improve assembly results. We validate our method by applying it to a series of 5 Mb simulation genomes derived from both mammalian and bacterial references. The results of applying our method to this simulation data are presented along with a discussion of the benefits and drawbacks of this technique.

## Introduction

Within a species, individual genomes differ from one another by a certain amount of genetic variation. These variations exist at different scales, ranging from single nucleotide variants (SNVs), to small-scale insertions and deletions (indels), up to large structural variations (SVs) of kilo- to mega-base scale. Many studies in genomics are focused on characterizing the content of these variations and identifying associations with diseases or other phenotypes [1,2]. While SNVs have been widely studied in recent years, larger-scale structural variations have been more dificult to characterize. Despite this, studies have shown a strong correlation between SVs and genetic disorders, including Crohn's disease and Down's syndrome [3-5].

In recent years, the development of high-throughput sequencing (HTS) technologies has made it possible to sequence an individual genome rapidly and at low cost. However, the problem of how to interpret this sequencing data remains. Traditionally, one of two approaches is taken. In *de novo *assembly, we consider the target (or donor) genome in isolation, using no information from prior assemblies. The output of a *de novo *assembly is a set of short sub-sequences, or contigs, representing the donor genome. Modern *de novo *assemblers typically employ a de Bruijn graph [6,7], and may take advantage of additional information from paired-end sequencing data [8,9] or multiple sequencing technologies [8] to join contigs into longer sequences.

*De novo *assembly contrasts with the alternative approach of resequencing. In this approach, we assume that the donor genome differs only by SNVs and indels from some reference genome. Resequencing, also known as read-mapping, takes advantage of the reference genome to map the sequencing reads to some position on the reference and identify the variations from the consensus of all mapped reads. Recent implementations of resequencing algorithms may also utilize paired-end sequencing data to disambiguate reads that map to multiple locations on the reference genome [10-12]. Some information derived from the read mappings, such as discordant read pairs (paired-end reads which map to conflicting positions on the reference), may be employed to detect the presence and content of structural variation. This is discussed further below.

It is helpful to consider these two approaches, *de novo *assembly and resequencing, in terms of their prior assumptions. *De novo *assembly methods assume no prior knowledge of the genome being assembled and instead treat each genome as if it represents a novel organism. Conversely, resequencing techniques assume the existance of only small variations from the reference genome. In many cases, both of these assumptions may be unrealistic. In particular, many genomic studies are focused on identifying the differences between genomes which are largely similar, but which may also contain large structural variations. For example, it is estimated that between two human individuals the total genetic variation may be as much as 8 Mb of sequence content [13]. In such cases neither *de novo *assembly nor resequencing adequately capture the correct assumptions and as a result may fail to identify the full range of variations present in the sequencing data. In particular, *de novo *assemblers generate large number of contigs, and provide little information about their relative ordering in the genome, making them unsuitable for identifying specific variations between individuals. Resequencing algorithms work very well for identifying SNVs in unique (non-repeat) regions of the genome that are largely conserved between the donor and the reference, but do not provide information on the larger structural variations.

To address this problem, a number of methods have been developed to both identify the loci of larger SVs [14-16] and estimate their content. These methods can be thought of as post-processing steps that take as input the data produced in resequencing and apply additional computation to those sequencing reads which are not consistent with the resequencing assumptions. Different methods exist, some focusing on characterizing *copy-number *variations (CNVs) [17], while others focus on large-scale insertions and deletions [18,19]. We believe these methods are limited in two key ways. First, they rely heavily on the results of resequencing, which can be unreliable in the presence of repeats, translocations, and inversions. Second, the methods tend to be highly specialized, focusing on a single type of mutation. Obtaining a complete picture of the genome thus remains difficult.

Because of this difficulty, many studies continue to rely on *de novo *assembly, even for organisms for which a high-quality reference exists. This is both computationally inefficient, typically requiring more than 100 GB of memory to compute, as well as undesirable in its results, which while unbiased, do not leverage the prior work that has been done in creating high-quality reference sequences.

A number of software packages have been developed in recent years with the aim of utilizing a set of reference genomes to produce a more optimized scaffolding, or layout, of the contigs produced in *de novo *assembly. OSLay [20] uses a maximum-weight matching algorithm to identify likely neighboring contigs. Treecat [21] builds a fully connected graph of the contigs, with edges weighted by the distance between syntenic regions in the reference, and attempts to find a minimum-weight Hamiltonian path through the graph using a greedy heuristic. Finally, PGA [22] uses a genetic algorithm to search the space of possible contig orderings. By relying on the contigs produced through *de novo *assembly, however, these methods may not take full advantage of the reference genome.

Our aim in this paper is to propose a novel model for the assembly of a donor genome which uses the reference as a guide, and to show how this approach improves assembly results over pure *de novo *assembly. Towards this goal, we formulate a novel graph construction capturing the similarities between the two genomes, and present the genome reassembly problem as a means of finding the valid set of paths through this graph. We present the results of our work on simulation data generated from both mammalian and bacterial genomes, and discuss the benefits and challenges of applying our method.

## Results

Here we present the results of our work, beginning with a brief overview of our method. We follow this with a discussion of our simulation results and the implications for the feasibility of our method.

### Method overview

**Figure 1:**
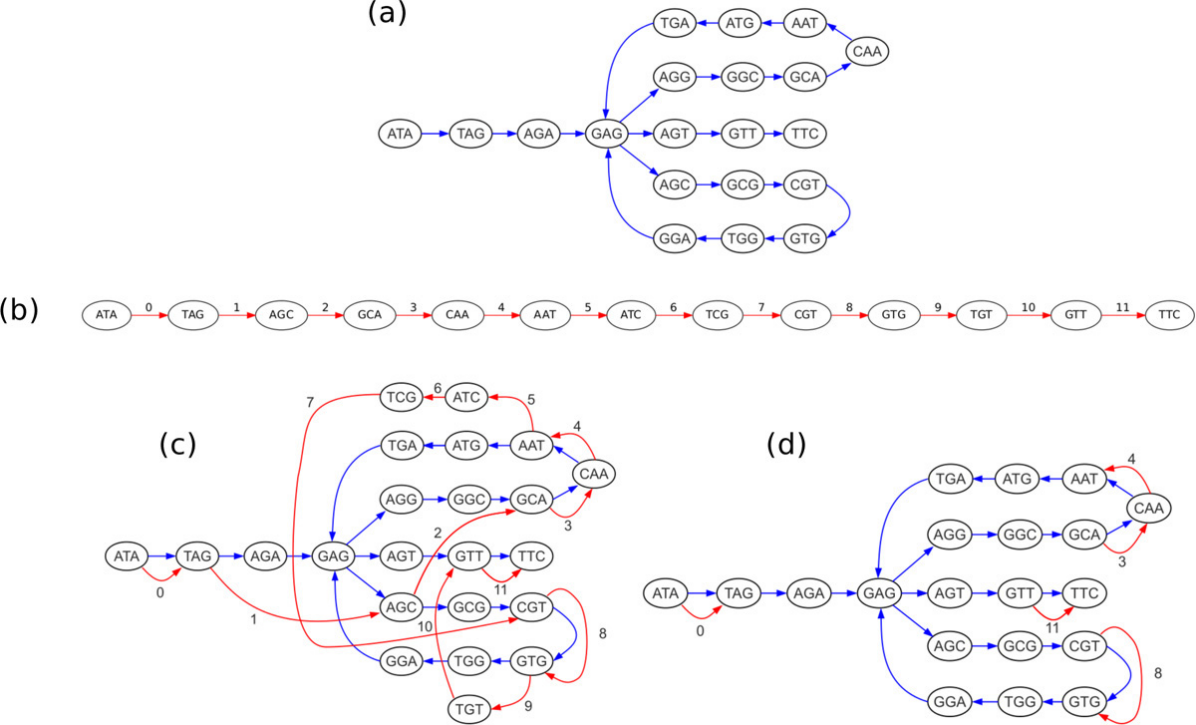
**A motivating example demonstrating how the use of a reference can help discover the most parsimonious traversal of the de Bruijn graph**. (a) de Bruijn graph of the donor sequence "ATAGAGGCAATGAGCGTGGAGTTC". (b) de Bruijn graph of the reference sequence "ATAGCAATCGTGTTC", including edge index labels. (c) Graph combining the donor and reference sequences. (d) Graph stripped of red edges with no parallel blue edge.

As an example of how a reference sequence can aid in assembly, consider the de Bruijn graph of a donor genome "ATAGAGGCAATGAGCGTGGAGTTC" in Figure 1a. Note that this graph has two possible Eulerian tours, one in which the lower branch is taken first, and one in which the upper branch is taken first. Only one of these tours spells the original donor genome, and a *de novo *assembly can not distiguish between them. If, however, we are given the reference sequence "ATAGCAATCGTGTTC," then it may be possible to discerne the correct tour. Figure 1c depicts the original de Bruijn graph augmented with the reference sequence, represented by red edges. In this new graph, it is now possible to choose the tour that is most *parsimonious *with the reference sequence. Stripping away the red edges that have no parallel blue edge, as shown in Figure 1d makes this more clear. We can now see that the tour following the upper branch first touches the reference edges 0, 3, 4, 8, and 11 in sequential order, with novel content in between. This indicates that the donor genome spelled by this tour represents a donor with only three insertions relative to the reference. The tour following the lower branch first touches the reference edges out of order in the sequence 0-8-3-4-11, indicating three insertions as well as a translocation. By appealing to parsimony, we can therefore conclude that the tour taking the upper branch first is the more likely of the two.

With this idea in mind, our method begins by building a graph of the contigs in the donor sequence. The construction of these contigs is flexible, and they may be derived from the sequencing reads through either *de novo *assembly or a hybrid process using both resequencing and assembly. Similar to the example above, in which parallel red edges were used to indicate local alignment between the donor and reference sequences, here each contig is compared to the reference genome and annotated with information denoting the local alignments of the contig to the reference. Our goal now is to find a tour of the graph, which corresponds to an ordering of the contigs, such that the size of "gaps" between aligned subsequences is limited by some value *τ*, which will be a parameter to our method. The problems with generating such a tour are two-fold. First, there may be spurious alignments caused by repeat or translocated regions, which will confound naïve attempts to traverse the graph. Second, there may be contigs with no alignments, representing sequences that are novel in the donor genome. We resolve these problems through a two-phased process of propagation and pruning, in which each contig first receives the set of alignments at contigs that are reachable within a distance of *τ*, then progressively eliminates spurious alignments by attempting to match them against alignments in adjacent contigs. The result is a graph in which much of the desired tour can be logically determined by inspection of the remaining alignment annotations. In those cases where the contig order can be determined, the adjacent contigs are merged as a means of simplifying the graph and promoting further elimination of spurious alignments.

It is important to note that while we use the alignment information in our method, it is never assumed that any specific alignment is correct. We believe this is a strength compared to other methods that more heavily rely on read mapping and as a result may be more biased towards the reference.

### Simulation results

In order to validate our method, we design a simulation framework using two reference genomes; the O157:H7 strain of the *E. coli* bacterial genome (NCBI NC011353.1), and chromosome 1 of the reference mouse genome (NCBIM37). The E. coli genome, at roughly 5 Mb in length, is used in its entirety, while we generate a simulation reference from the mouse genome by sampling a 5 Mb subsequence from the chromosome.

For each reference genome, we generate simulated donor genomes by applying a series of mutations to the reference, including insertions, deletions, duplications, and translocations. We vary the average size of the mutation events from 5 Kb to 50 Kb, such that these events comprise roughly 15% of donor genome. We further apply a set of SNV mutations at a rate of 0.1%.

We generate simulated paired-end sequencing data from each donor genome using a read length of 100 bp and fixed insert size of 500 bp. In all cases we assume error-free reads and uniform coverage (a read from every position). While these assumptions are unrealistic in practice, correcting for read errors and variable coverage are orthogonal problems which have been studied independently [23-25]. Although our method will need to be extended to account for the types of data encountered in real-world studies, we believe these preliminary results show significant promise.

For each data set, we perform paired-end assembly using Velvet [9] as a performance baseline, using a *k*-mer size of 99 bp. We then apply our own method and demonstrate that we are able to acheive significant improvements in both the number and size of assembled contigs. We validate that our contigs remain accurate by aligning them back against the simulation donor genome and observe that on average fewer than 1% are misassembled. This indicates that our method is relatively conservative, and does not bias excessively towards the reference. Refer to Table 1 for the results of our experiments on three different simulated donor sequences derived from the mouse reference.

We further evaluate our method by comparing two different strains of the E. coli bacteria, using the O157:H7 strain as a reference, and the K-12 strain as a donor. The K-12 strain is significantly shorter in length than the O157 strain, indicating the presence of large-scale deletions. This is supported by our assembly results, which indicate mutation events up to 120 Kb in size. The full results of our simulations on the E. coli reference are reported in Table 2.

**Table 1:** Results of running both Velvet and our method on simulated mouse chromosomes.

	Velvet			Our method			
**Donor genome**	**# Contigs**	**N50**	**Max contig**	**# Contigs**	**N50**	**Max contig**	**Accuracy**

Mouse, 5 Kb	1014	14315	56677	**352**	**73042**	**288172**	**99.7%**
Mouse, 25 Kb	773	19038	102858	**386**	**88473**	**227406**	**99.7%**
Mouse, 50 Kb	705	21721	98684	**410**	**117127**	**336208**	**99.2%**

Each simulated chromosome is 5 Mb in length, containing roughly 15% mutated content, using mutation event sizes of 5, 25, and 50 kb. In each case, contig statistics are given for the Velvet assembly and for our method, along with the accuracy of our computed contigs. Accuracy is calculated as the percentage of computed contigs that align back to the donor genome.

**Table 2:** Results of running Velvet and our method on *E.coli*-based genomes.

	Velvet			Our method			
**Donor genome**	**# Contigs**	**N50**	**Max contig**	**# Contigs**	**N50**	**Max contig**	**Accuracy**

*E. Coli* O157, 5 Kb	1034	25477	158013	**422**	**56750**	**274293**	**99.5%**
*E. Coli *O157, 25 Kb	870	71194	286061	**727**	**96535**	**285958**	**99.6%**
*E. Coli* K12	166	125649	327149	**33**	**429486**	**734812**	**97.0%**

In the first three cases, simulated donor genomes are derived from the O157 reference by applying a series of mutations (insertions, deletions, and SNVs). In the final case, the K12 *E. coli* strain is used as a donor and compared against the O157 reference.

It is important to note that while comparisons against *de novo *assemblers such as Velvet provide a valuable baseline for performance metrics, our method incorporates a significant source of additional information (the reference genome). Direct comparisons are therefore inherently unfair. Our results are instead intended to show the possible extent to which *de novo *results could be improved upon through the incorporation of existing reference sequences and reasonable assumptions.

## Methods

Let *R *be a reference genome and *D *be our donor genome. We define *S_R _*and *S_D _*to be multisets (allowing repeats) of subsequences of the reference and donor genomes, respectively. In all cases, *S_R _*represents the spectrum of *k*-mers, a single *k*-mer sampled from every position in *R*. *S_D _*is dependent on our sequencing technology but in all cases is an approximation of the spectrum. We proceed first by defining a series of graph constructions that facilitate our method, then describe a message-passing formulation of our method that is concise and simple to implement.

### Reference/donor graphs

Given the multisets of *k*-mers *S_R _*and *S_D_*, we can construct de Bruijn graphs *G_R _*= {*V_R_, E_R_*} and *G_D _*= {*V_D_, E_D_*}, where *V_R _*(*V_D_*) is the union of all (*k *- 1)-mers in *S_R _*(*S_D_*) and *E_R _*(*E_D_*) is the multiset sum of all *k*-mers in *S_R _*(*S_D_*). In more simple terms, we are given a set of *k*-mers, either sampled directly from the reference or generated by breaking up reads of length *l *in the donor sequencing data into *l *- *k *+ 1 *k*-mers, and we construct a graph such that every *k*-mer is represented by an edge. Because we are interested in the similarities beteween the donor and the reference, it is helpful to combine both the reference and the donor in a single graph as described below.

**Definition: **a **reference/donor graph ***G_RD _*is the superposition of de-Bruijn graphs *G_R _*and *G_D _*such that , where the  operator indicates multiset sum. In order to maintain distinction between reference and donor edges, we assign an edge color of red to *E_R _*and blue to *E_D _*in the construction of *G_RD_*. Each reference edge *e *∈ *E_R _*is annotated with an integer index *e*.*pos *equal to the corresponding position of the edge in the reference genome.

**Figure 2:**
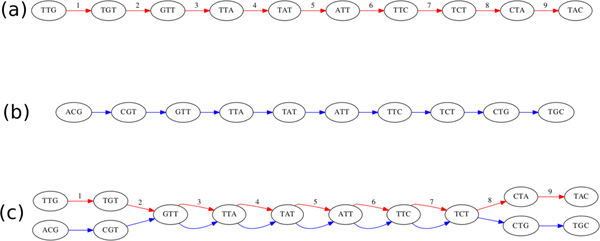
**A most basic example of a reference/donor graph, constructed from the superposition of the two original graphs**. (a) Reference graph *G_R_*. (b) Donor graph *G_D_*. (c) The graph representing the reference sequence "TTGTTATTCTAC" and donor sequence 'ACGTTATTCTGT'. The donor subsequence "GTTATTCT" is reference-parallel, with reference marker *R*[3: 7], indicating that it aligns to position 3-7 in the reference sequence.

Refer to Figure 2 for a simple example of such a graph. While we are interested in constructing tours of this reference/donor graph, the traditional definition of an Euler tour in which each edge is visited exactly once no longer applies. We are only intersted in tours which exclusively touch donor edges, as these are the only tours that represent the sequencing data. This leads to the following simple definition.

**Definition: **a **donor tour **of a reference/donor graph is a complete tour that includes only blue (donor) edges.

In other words, a donor tour of *G_RD _*is equivalent to an Euler tour of *G_D_*.

We are now interested in a concise way to characterize the similarities between the reference and the donor. We start by considering those cases in which a red and blue edge are parallel in the graph (denoted by the || operator). The notation and terminology we will use in discussing these cases is defined as follows.

**Definition: **a donor edge *e ***∈ ***G_RD _*is considered **reference-parallel **if there exists a reference edge *R*(*e*) ∈ *G_RD _*such that *R*(*e*) || *e*. A sequence of donor edges *E *= {*e*_1_, *e*_2_, . . ., *e_n_*} is considered reference-parallel if for every pair of consecutive edges *e_i_, e_i_*_+1 _∈ *E *there exist parallel reference edges *R*(*e_i_*), *R*(*e_j_*) ∈ *G_RD _*such that *R*(*e_j_*).*pos *= *R*(*e_i_*).*pos *+ 1. A sequence of donor edges *E *= {*e*_1_, *e*_2_, . . ., *e_n_*} is considered **novel **if it is not reference-parallel.

**Definition: **the reference indexes of a reference-parallel sequence are the values *R*(*e*_1_).*pos, R*(*e*_2_).*pos*, . . ., *R*(*e_n_*).*pos *and are concisely represented as the pair *m *= (*R*(*e*_1_).*pos, R*(*e_n_*).*pos*), referred to as a **reference marker**. The beginning and end of the reference-parallel sequence are referred to as *m.start *and *m.end*, respectively. Given an edge *e*, the set of all reference markers associated with the edge is denoted *markers*(*e*).

Refer to Figure 2c for an example of a complete reference/donor graph with a reference-parallel sub-sequence, along with the associated reference marker. Having defined this notion of reference markers, it is natural to consider the associations *between *them. In particular, we are interested in pairs of reference markers that are within a certain distance in the graph, and which represent nearby segments of the reference genome.

**Definition: **given two reference markers *m_i _*and *m_j_*, we say that *m_i _***connects to ***m_j _*within distance *d *if *m_j_.start *- *m_i_.end *<*d *and the markers are separated by at most *d *edges in the graph. This relationship is indicated by $m_{i}\overset{d}{\to }m_{j}$.

With this graph construction, we can now de ne the genome reassembly problem.

**The genome reassembly problem**: given a reference/donor graph *G_RD_*, generate a donor tour of the graph which maximizes the summed lengths of all reference-parallel subsequences.

We note here that this is an extremely large combinatorial problem, and as such a solution is impractical. We therefore formulate a new problem, imposing an assumption on the size of any single variation event in the donor genome.

**The *τ*-gap genome reassembly problem**: given a reference/donor graph *G_RD_*, generate a donor tour of the graph such that for any novel subsequence *X *of the tour, |*X*| <*τ *and *X *is bounded by reference-parallel subsequences with reference markers at most *τ *apart.

### Condensed reference/donor graph

**Figure 3:**
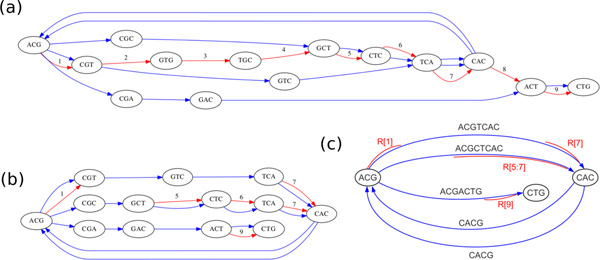
**Construction of a condensed reference/donor graph in a more complex case**. In the first pass, any reference edges with no parallel donor edge are removed. In the second pass, linear subpaths are condensed to single edges, and the parallel reference edges are summarized using reference markers. (a) Initial reference/donor graph. (b) Intermediate graph with isolated reference edges removed. (c) Final condensed reference/donor graph, with reference markers shown.

In order to reduce the computation and storage demands of the algorithm, we first transform the reference/donor graph *G_RD _*into a *condensed *reference/donor graph *G_CRD_*. The condensed graph is produced by concatenating linear (non-branching) sequences of donor edges into single edges representing a contig. Rather than storing the reference edges, we store only the reference markers associated with any reference-parallel subsequences in a given contig. Refer to Figure 3 for an example of a condensed graph. Note that while each edge in the reference/donor graph had an implicit edge length of 1, each edge in the condensed graph has a length equal to the length of its contig.

With the condensed reference/donor graph in mind, we may think of a valid traversal as one which touches a sequence of reference markers, one from each edge in the path. For this traversal to be valid, each adjacent pair of reference markers in this sequence must be separated by a distance of at most *τ*. We can therefore encode the set of valid traversals by maintaining the list of reference markers attached to each edge, and pose the problem as follows.

**The *τ*-gap genome reassembly problem on a condensed reference/donor graph**: given a condensed reference/donor graph *G_CRD_*, generate a tour of the graph consisting of a sequence of edges *T *= {*e*_1_, *e*_2_, . . ., *e_m_*}, such that for every adjacent pair of edges *e_i_, e_i_*_+1_, there exist reference markers *m_i _*∈ *markers*(*e_i_*), *m_j _*∈ *markers*(*e_i_*_+1_) with *m_j_*.*start *- *m_i_*.*end *≤ *τ*.

Note, however, that this formulation requires at least one reference marker at *every *edge in the graph. Initially, the graph will likely not satisfy this requirement, as there will be many edges which have no analogue in the reference. This is not an indication that our assumption has been violated, but simply that these edges carry no information. We resolve this problem through a method referred to as *marker propagation*, which aims to update the reference markers at each edge with information from neighboring edges.

### Message passing algorithm

**Figure 4:**
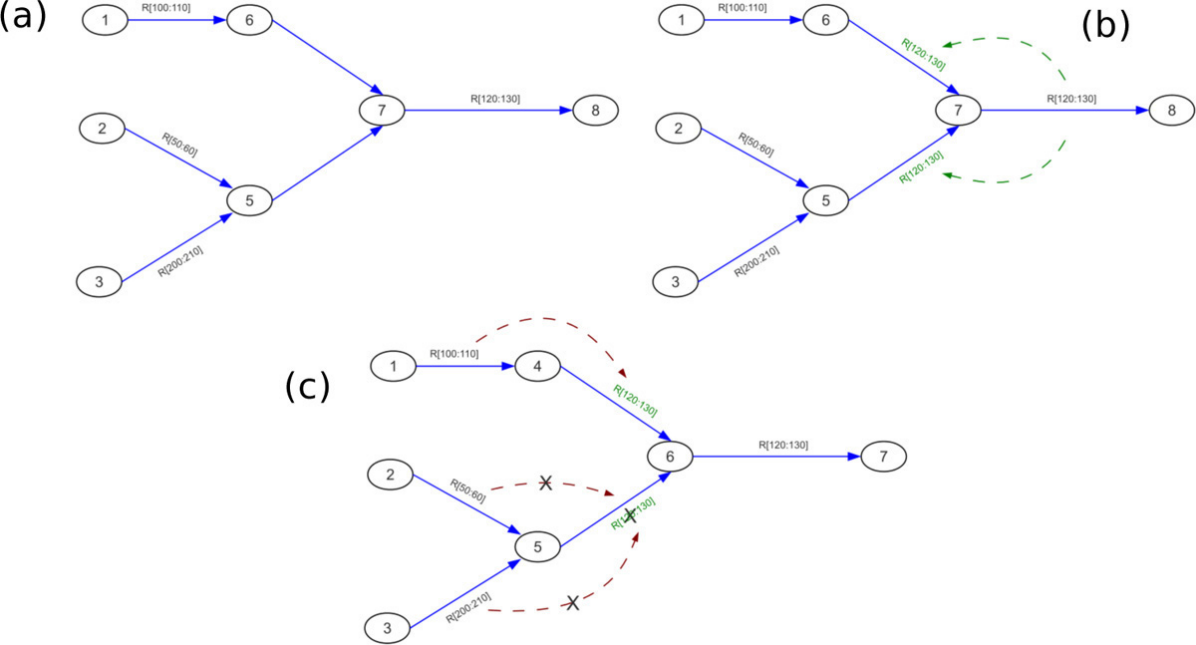
**Showing the process of propagation and pruning, assuming *τ *= 15**. All edges shown have an edge length of 10. Initially, we have the graph in (a), in which the edges (6,7) and (5,7) have no reference markers. The marker attached to edge (7,8) is propagated along incoming edges up to a distance of *τ*, resulting in the graph shown in (b). Note that the marker has been added to edges on both the top and bottom branch. Finally, in the pruning phase, each marker is checked for connectedness. On the top branch, the newly added marker is connected, while on the bottom branch, no adjacent marker connects, so the marker is pruned.

Having constructed the reference/donor graph, our aim is to encode within the graph exactly those traversals which satisfy our initial assumptions on *τ*, the maximum size of any variation event. Specifically, we would like to know for any pair of adjacent edges, whether a path through the two edges is valid. In order to know this, we must know not only what reference markers exist at a given contig, but also the set of markers at contigs reachable along some directed path within a distance of *τ*. We solve this problem by propagating information along edges in the graph. This process leaves many spurious markers, however, so we then apply a pruning phase to eliminate these. Refer to Figure 4 for an example.

#### Propagation

As previously described, each edge in the condensed reference/donor graph stores a list of the reference markers associated with any reference-parallel subsequences within that edge's contig. The first phase of the message-passing algorithm propagates this information throughout the graph, such that each edge additionally stores a list of reference markers at edges that are *reachable *along directed edges in the graph, within a distance of *τ*. While there are different methods for computing this list, we provide the message-passing formulation here as the most concise.

A message in this propagation phase consists of a set of pairs, each pair (*m, d*), consisting of a reference marker *m *and distance *d *(in terms of total edge length) that the marker has been propagated so far. On receiving a message, an edge *e *checks each pair in the set, incrementing the distance values by *e.length*. Any pairs with *d *>*τ*, or which have already been added to the list, are eliminated, while the rest are stored in the edge's local list and then propagated on as new messages to each incoming neighbor. If no markers from an incoming message are added to the local list, no new messages are generated. The propagation phase ends when there are no messages remaining in the graph. Algorithm 1 demonstrates the message handler for the propagation phase.

**Algorithm 1 **Propagation_ReceiveMessage(edge, message)

1: *added *← ∅

2: **for **(*m, d*) **∈ **message **do**

3:   *d *← *d *+ edge.*length*

4:   **if ***d *<*τ ***then**

5:     edge.*distant_markers *← edge.*distant*_*markers *∪ *m*

6:     *added *← *added *∪ (*m, d*)

7:   **end if**

8: **end for**

9: **if ***added *≠ ∅ **then**

10:     *Propagation_SendMessage*(*in_neighbors*(*edge*), *added*)

11: **end if**

#### Pruning

Following the propagation phase, each edge in the graph must have at least one reference marker in its list (if any edge does not, then our assumption on *τ *has been violated). There will also be many edges which have *excess *reference markers. That is, reference markers which are touched by no valid tour of the graph, yet which may confound or complicate our attempts to generate such tours. We iteratively prune these excess markers through a second message-passing phase.

In the second phase, each edge on receiving a message inspects the markers in its list, categorizing each as either "connected" or " orphaned." A connected marker *m *is one for which there are associated markers *m_in _*and *m_out_*, belonging to some incoming and outgoing edge, respectively, such that $m_{in}\overset{\tau }{\to }m\overset{\tau }{\to }m_{out}$. An orphaned marker is any marker that is not connected, and each orphaned marker is removed from the edge's list. Whenever an edge removes some reference marker from its list, a message is sent to each neighboring edge. The phase begins by sending a message to each edge in the graph, and ends when there are no messages remaining. Algorithm 2 provides a more concrete example of a message handler for this phase.

**Algorithm 2 **Pruning_ReceiveMessage(edge)

1: r*emoved *← ∅

2: **for ***m_e _*∈ edge.*markers ***do**

3:   *connected *← **false**

4:   **for ***outgoing ***∈ ***outgoing_neighbors *(edge) **do**

5:     **for ***m_o _*∈ *outgoing.markers ***do**

6:       **if ***m_e _*$\overset{\tau }{\to }$*m_o _***then**

7:         *connected *← **true**

8:       **end if**

9:     **end for**

10:   **end for**

11:   **if **¬ *connected ***then**

12:     edge.*markers *= edge.*markers*/*m_e_*

13:     *removed *← *removed *∪ *m_e_*

14:   **end if**

15: **end for**

16: **if ***removed *≠ ∅ **then**

17:   *Pruning_SendMessage*(*neighbors*(*edge*))

18: **end if**

#### Merging and iteration

**Figure 5:**
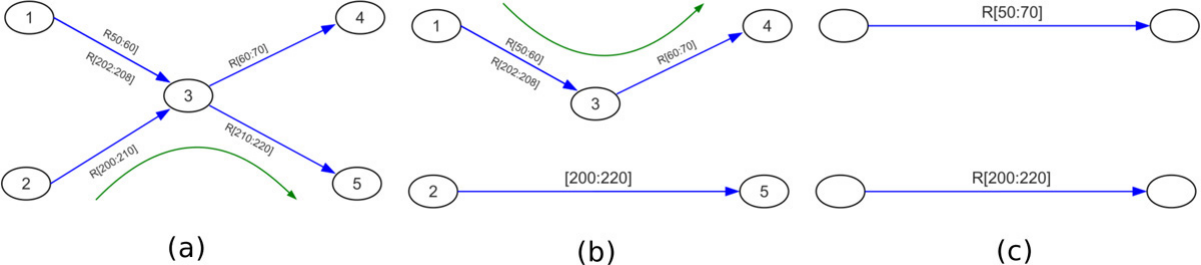
**Showing the process of merging edges when there is only one possible path**. In (a), edge (1,3) could be followed by either (3,4) or (3,5) based on the reference markers. Edge (2,3) however can only be followed by (3,5). In (b), (2,3) and (3,5) are merged, which makes it possible to eliminate a marker from edge (1,3), and to merge it with (3,4). (c) shows the final state.

During the pruning phase, we will observe many cases in which there is only one possible path for a traversal to follow. In these cases, we can merge the adjacent edges and recompute their respective markers. Each such merge operation will reduce complexity of the graph, further allowing reference markers to be eliminated. Refer to Figure 5 for an example. Each merge therefore produces a new round of pruning, and this repeats iteratively until nothing more can be done.

### Implementation notes

The condensed reference/donor graph is an annotated version of the condensed de-Bruijn graph, and can be easily constructed as such. In recent years, a number of methods have been proposed to construct the condensed de-Bruijn (contig) graph. The simplest method was given in [6] and constructs a de-Bruijn graph using single reads (no paired-end information). This method has since been extended to incorporate paired-end [26], and number of available assemblers implement some variation on this idea [8,9,27]. For our purposes, any method is sufficient, though methods which produce longer contigs are obviously desirable. Methods which utilize the reference in assembly may also be used, provided they generate overlapping contigs which form a graph. In our implementation, we construct the contig graph following the method in [28] using paired-end information, and find local alignments using a custom method based on the read-mapping tool BWA [11]. Other tools such as BLAST [29] may be used to compute the alignments.

Our simulations were performed using a single-threaded implementation running on a 3.2 GHz processor with 16 GB of memory, and demonstrated a worst-case running time of approximately 1 hour. The time complexity of the algorithm is *O*(*n*^3^) in the worst case, where *n *is the number of contigs. This is driven by an initial computation of the all-pairs shortest distances, but in general this can be highly optimized as we do not care about distances larger than *τ*. Each phase of the algorithm can also be parallelized with minimal changes.

## Discussion

The goal of any genome sequencing project is to characterize the full genomic content of an individual organism. With the steeply declining cost of genome sequencing in recent years, there has been significant focus on new and improved methods for both *de novo *assembly and resequencing. Despite this focus, however, there have been few methods developed to bridge the gap between these areas. While progress has been made in discovery and assembly of structural variation, the tools remain highly specialized. In this study, we proposed a novel graph construction that concisely represents the similarities between a reference and donor genome, and developed a method using the graph to disambiguate contig ordering. Through simulation, we demonstrated that this method can be effective when working with related bacterial genomes, but significant challenges remain.

One such challenge is that as input to our method we require an estimate of the maximum mutation event size. In practice, this value is not known, and this is currently a significant drawback of our method. It is possible, however, that methods could be developed to estimate this parameter. For example, iterative application of our method with successively larger or smaller values could help discover the true maximum size. Alternatively, the parameter could be estimated directly from the alignment data. Notably, we have also not discussed the application of our method in the presence of read errors. The effect of these data imperfections can be mitigated to an extent by the application of preprocessing methods to correct the errors prior to assembly. Recent studies have shown that read errors can be significantly reduced even in the presence of non-uniform coverage [24,25]. However, further experiments should be performed to validate the effectiveness of our method under less ideal conditions.

Despite these remaining challenges, we believe our method presents a novel approach to the challenge of genome assembly that takes advantage of the increasing availability of reference sequences. It is our hope that this work can help motivate future research into unified reassembly methods.

## Competing interests

The authors declare that they have no competing interests.

## Authors' contributions

N.P. developed and implemented the algorithm, generated and analyzed results, and authored this manuscript. B.S. and E.E. provided the initial problem framework and algorithmic approach, insights and suggestions on the development of the algorithm, and critiques of the manuscript.

## Declarations

The publication costs for this article were funded by the corresponding author's institution.

This article has been published as part of *BMC Genomics *Volume 14 Supplement 1, 2013: Selected articles from the Eleventh Asia Pacific Bioinformatics Conference (APBC 2013): Genomics. The full contents of the supplement are available online at http://www.biomedcentral.com/bmcgenomics/supplements/14/S1.
